# Association of VTN Genotype with Plasminogen Activator Inhibitor-1 Activity in Late-Onset Alzheimer’s Disease

**DOI:** 10.3390/genes17050516

**Published:** 2026-04-27

**Authors:** Deniz Agirbasli, Mehmet Agirbasli, Mehmet Emin Cakir, Meltem Muftuoglu

**Affiliations:** 1 Department of Medical Genetics, Cerrahpasa Faculty of Medicine, Istanbul University-Cerrahpasa, 34098 Istanbul, Turkey; deniz.agirbasli@iuc.edu.tr; 2 Department of Cardiology, School of Medicine, Istanbul Medeniyet University, Uskudar, 34700 Istanbul, Turkey; agirbasli@gmail.com; 3 Department of Neurology, Sancaktepe Şehit Prof. Dr. Ilhan Varank Training and Research Hospital, Sancaktepe, 34785 Istanbul, Turkey; dr.emincakir@hotmail.com; 4 Department of Neurology, Goztepe Training and Research Hospital, Istanbul Medeniyet University, Kadikoy, 34722 Istanbul, Turkey; 5 Department of Molecular Biology and Genetics, Faculty of Engineering and Natural Sciences, Graduate School of Natural and Applied Sciences, Acibadem Mehmet Ali Aydinlar University, Atasehir, 34752 Istanbul, Turkey

**Keywords:** Late-onset Alzheimer’s Disease, plasminogen activator inhibitor-1, vitronectin, Serpine1, APOE

## Abstract

Background/Objectives: Late-onset Alzheimer’s disease (LOAD) is a multifactorial neurodegenerative disorder involving the interaction of genetic and environmental factors. Dysregulation of the fibrinolytic system, particularly an increase in plasminogen activator inhibitor-1 (PAI-1) levels, may contribute to Alzheimer’s pathology. Vitronectin (VTN) regulates fibrinolysis by stabilizing PAI-1. This study investigated the relationships between plasma PAI-1 activity and *VTN*, *SERPINE1* (*PAI-1*), and *APOE* gene variants in nineteen LOAD patients (>65 years) and ten cognitively normal age-matched control groups. Methods: Targeted next-generation sequencing was used to analyze the *VTN*, *APOE*, and *SERPINE1* genes in 19 LOAD patients and ten controls. Additionally, plasma PAI-1 activity was measured in both groups. Results: Plasma PAI-1 activity was statistically significantly higher in LOAD patients compared to controls (*p* = 0.04). Targeted next-generation sequencing results showed that *VTN* 5′-UTR variants (rs7212814, rs1555584131, rs71135830, and rs11437594) were found in all patients and observed in 20% of controls (*p* = 0.0001). The *VTN* rs704 variant was detected in 84% of patients and 29% of controls (*p* = 0.001). *VTN* 5′-UTR variants showed Spearman correlation with PAI-1 activity (r = 1.0; *p* < 0.0001). *SERPINE1* 3′-UTR variants (rs11178, rs41423845) were found to be associated with the disease (*p* = 0.027; *p* = 0.0001). The *APOE* *ε3*/*ε4* genotype was present in 52.6% of patients and was not associated with PAI-1 activity. *VTN* variants showed an association with LOAD. Conclusions: These findings suggest that *VTN* variants may contribute to LOAD pathogenesis by affecting PAI-1 and leading to fibrinolytic system dysregulation.

## 1. Introduction

Alzheimer’s disease (AD) is a progressive neurodegenerative disorder characterized by multifactorial pathophysiology. Early-onset AD, which typically manifests before age 65, has a strong genetic basis and follows a Mendelian dominant inheritance pattern. Additional genetic variants may influence the age of onset and disease progression. The pathophysiology of late-onset AD (LOAD) includes age-related damage. The ideal age cut-off for defining LOAD depends on genetic variability [1]. The deposition of amyloid fibrils and neurofibrillary tangles has been implicated in the pathophysiology of AD [2]. The impaired fibrinolytic system fails to clear fibrin deposits, leading to the abnormal accumulation of fibrin(ogen) in the AD brain [3,4].

A key regulator of fibrinolysis is plasminogen activator inhibitor-1 (PAI-1). PAI-1 is a serine proteinase inhibitor (serpin family member) that inhibits tissue- and urokinase-type plasminogen activators (t-PA and u-PA) [5]. It is a marker and mediator in several biological processes, including thrombosis, angiogenesis, fibrosis, connective tissue diseases, malignancy, and metastasis [6]. It has three conformations, including free active PAI-1, inactive PAI-1 complexed with t-PA, and latent PAI-1 (an inactive PAI-1 conformation) [7,8]. The inhibitory activity and stability of PAI-1 are critically dependent on its conformational flexibility and its interaction with binding partners [9]. Notably, the extracellular matrix glycoprotein vitronectin (VTN) plays a significant role in the modulation of fibrinolytic system activity by increasing the stability of PAI-1 [10,11]. Active PAI-1 rapidly binds to t-PA, whereas the clearance of active t-PA and PAI-1 is faster than the PAI-1/t-PA complex [12]. The polymorphism rs704C>T in the *VTN* gene alters its protein expression and functionality and has been associated with age-related macular degeneration [13,14,15], highlighting the importance of VTN in age-related pathologies. Elevated PAI-1 activity reduces plasmin generation, and plasmin is known to degrade amyloid-β peptides, a key pathological hallmark of AD. Although VTN is known to bind to PAI-1 and affect its stability and activity, little is known about how VTN and PAI-1 interact in AD, the influence of genetic variants in the *VTN* and *SERPINE1* genes on this interaction, or how this interaction contributes to AD pathology. Given that VTN stabilizes active PAI-1 and modulates fibrinolytic activity—a process implicated in neurodegeneration—we hypothesized that genetic variants in *VTN* may influence PAI-1 activity and contribute to LOAD risk. To test this hypothesis, we analyzed *VTN* and *SERPINE1* variants and measured PAI-1 activity in a cohort of LOAD patients and cognitively healthy, age-matched controls. The *APOE* gene has been implicated in the pathophysiology of LOAD. *APOE* is polymorphic at two single nucleotides (rs429358 and rs7412), resulting in three different alleles (ε2, ε3, and ε4). While the ε4 allele of the *APOE* gene significantly increases LOAD risk, the ε2 allele is protective relative to the common ε3 allele [16]. This study also aims to elucidate a novel potential link between *VTN* and *APOE* genetics, PAI-1 biology, and the pathogenesis of LOAD.

## 2. Materials and Methods

### 2.1. Study Population

Peripheral blood samples were collected from 19 LOAD patients (>65 years) and 10 age-matched cognitively controls without any AD family history, recruited at the Department of Neurology, Medeniyet University Goztepe Training and Research Hospital, Istanbul, Turkey. Patients with acute illness and other forms of neurological diseases were excluded. The clinical diagnosis of LOAD was made according to the Neurological and Communicative Disorders and Stroke–Alzheimer’s Disease and Related Disorders Association (NINCDS-ADRDA) criteria and the criteria of the Diagnostic and Statistical Manual of Mental Disorders, 4th ed. (DSM-IV). Cognitively normal participants received the same assessment as the LOAD patients and were determined to be cognitively unimpaired. In addition to the clinical information, including age, gender, duration of disease, and age of onset, the cognitive status was assessed using the Mini-Mental State Examination (MMSE). Written informed consent was obtained from all subjects before their inclusion in the study. All procedures were performed in compliance with relevant laws and institutional guidelines. Acibadem University Institutional Review Board (ATADEK) approved the study protocol with decision number 2025-01/28.

### 2.2. PAI-1 Activity Measurement

Blood was collected in EDTA-coated tubes after overnight fasting. The plasma was separated by low-speed centrifugation at 4 °C right after venipuncture. The PAI-1 human chromogenic activity kit (ab108894) (Biotech, Life Sciences, Cambridge, UK) was employed to determine PAI-1 activity in plasma samples following the manufacturer’s instructions. In this assay, an excess amount of t-PA is added to undiluted plasma, allowing the formation of inactive PAI-1/t-PA complexes. Residual t-PA activity is then determined in a coupled reaction containing t-PA, plasminogen, and a plasmin-specific chromogenic substrate. The plasmin generated in this process cleaves a substrate to release para-nitroaniline (pNA), a yellow chromophore measured at 405 nm. The PAI-1 enzymatic activity presented was the average of three independent experiments.

### 2.3. Targeted Next-Generation Sequencing of VTN, APOE, and SERPINE1 Genes

Total DNA was isolated using a DNAeasy Blood & Tissue kit (Qiagen, Hilden, Germany) according to the manufacturer`s protocol. DNA quality and quantity were evaluated using Qubit dsDNA HS Assay Kit (Thermo Fisher Scientific, Waltham, MA, USA) according to the manufacturer’s protocol. Targeted next-generation sequencing (NGS) was performed using the Illumina platform in a commercial laboratory (Eurofins, Hamburg, Germany). FASTQ files were provided by Eurofins. Alignment of FASTQ reads to the GRCh37 (hg19) human reference genome and variant detection were performed using the Variant Caller plugin to generate VCF files. Comparative analyses of case and control datasets were conducted with CLC Genomics Workbench (v.9.0.1; Qiagen, Hilden, Germany). Statistically significant variants were determined based on Bonferroni-adjusted Fisher’s exact test *p*-values.

### 2.4. Statistical Analysis

Statistical analysis was performed using the GraphPad Prism 11 software. A two-tailed unpaired Student *t*-test was used to compare the PAI activity between samples and controls. The relationships among parameters were assessed using Pearson’s or Spearman’s correlation coefficient according to the normality of the data. Fisher’s exact test was performed to compare the allele frequencies between LOAD and controls. A *p*-value of <0.05 was considered statistically significant.

## 3. Results

### 3.1. Plasma PAI-1 Activity Levels Are Increased in LOAD Patients

**Figure 1:**
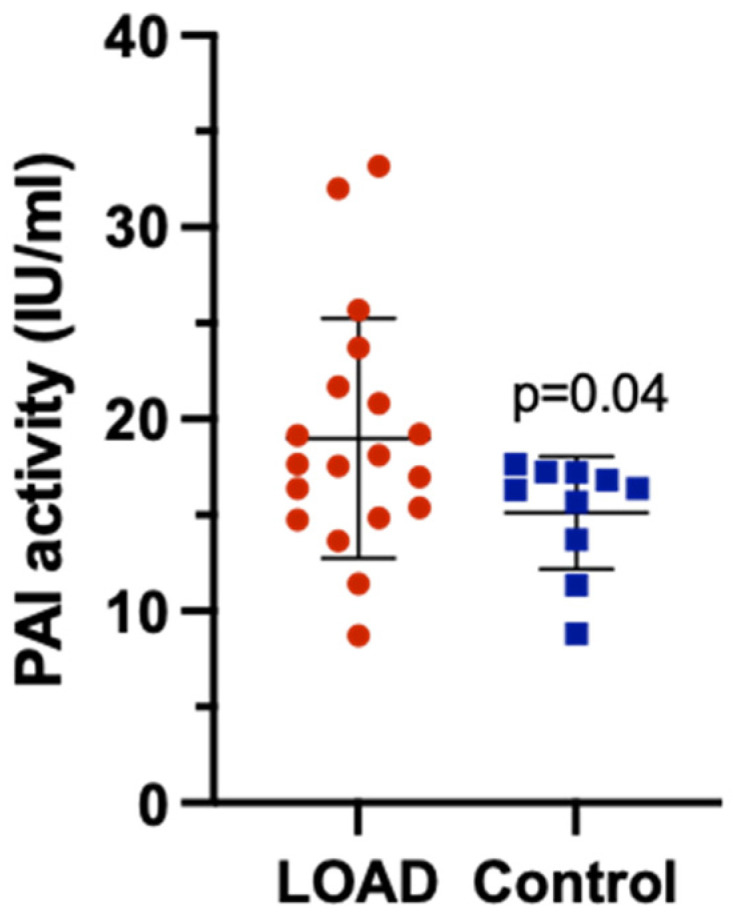
PAI-1 activity. A two-tailed *t*-test was used to compare the PAI activity between the LOAD samples and the control.

Since dysregulation of the fibrinolytic system, particularly elevated PAI-1, may contribute to Alzheimer’s pathology by reducing fibrin clearance, PAI-1 activity was measured in peripheral blood samples from 19 LOAD and 10 cognitively normal age-matched control subjects. The demographic and clinical characteristics of the participants are shown in Table 1. PAI-1 activity was statistically significantly increased in LOAD patients (18.89 ± 6.45 IU/mL) compared to the control group (15.12 ± 2.93 IU/mL) (*p* = 0.04) (Figure 1). No statistically significant correlation was found between PAI-1 activity and MMSI and CDR scores, or PAI-1 activity and age (*p* > 0.05). Although no statistically significant difference was observed across MMSE severity groups (*p* = 0.788), mean PAI-1 activity was numerically slightly lower in the severe group (17.5 ± 4.4 IU/mL, n = 4) compared to the mild (18.9 ± 8.7 IU/mL, n = 8) and moderate (20.1 ± 6.3 IU/mL, n = 7) groups. The standard deviations ranged from ±4.4 to ±8.7 IU/mL, indicating substantial inter-individual variability, which provided limited statistical power to detect small or moderate effect sizes.

We performed a sex-adjusted analysis using multiple linear regression with sex and group (patient/control) as independent variables and plasma PAI-1 activity as the dependent variable. After adjustment for sex, the difference between patients and controls was no longer statistically significant (β = 3.77, *p* = 0.09). Sex itself did not show a significant independent effect on PAI-1 activity (β = 0.20, *p* = 0.92). The overall model was not significant (R^2^ = 0.10, *p* > 0.05)

### 3.2. SERPINE1 (PAI-1), VTN, and APOE Gene Variants in LOAD Patients and Control Subjects

To investigate the genetic basis underlying not only the neuronal but also the vascular and coagulation components of LOAD, we performed targeted NGS for the *VTN*, *SERPINE1* (PAI-1), and *APOE* genes in peripheral blood samples from 19 Alzheimer’s patients and 10 cognitively normal, age-matched control subjects (Table 2). Two variants were identified in the 3′-UTR region of the *SERPINE1* gene, which encodes the PAI-1 protein: rs11178 (*p* = 0.027) and rs41423845 (*p* = 0.0001) (Table 2). Rs41423845 is a common (GnomAD = 0.87) synonymous polymorphism and is therefore not considered biologically statistically significant. The second *SERPINE1* 3′-UTR variant, rs11178 (*p* = 0.027), showed no statistically significant correlation with PAI-1 activity levels in variant carriers (*p* > 0.05). Two exonic variants in the *SERPINE1* gene, rs6090 and rs6092, were identified in LOAD patients (Table 2). The *SERPINE1* rs6092 variant was present in four of the 19 LOAD patients (21.05%). The mean PAI-1 activity was 23.18 ± 12.31 IU/mL in LOAD patients carrying this variant, compared to 16.70 ± 3.65 IU/mL in non-carriers.

Analysis of the *VTN* gene revealed numerous variants that showed statistically significant differences between LAOD patients and controls. Four variants in the 5′-UTR region of the *VTN* gene (rs7212814, rs1555584131, rs71135830, and rs11437594) were present in all 19 LOAD patients and in 2 out of 10 control subjects, while the remaining 7 controls carried none of these variants (Table 2; *p* = 0.0001). Additionally, an intron variant, rs2227725, was similarly detected in all patients (100%) and 20% of controls (*p* = 0.0001). Furthermore, the rs704 variant (p.Thr400Met) was observed in 16 out of 19 LOAD patients (84%) and in 2 out of 10 control subjects (29%) (Table 2; *p* = 0.001). The presence of *VTN* 5′-UTR variants showed a Spearman correlation with PAI-1 activity (Spearman’s r = 1.0, *p* < 0.0001), indicating an association between *VTN* genotype and PAI-1 levels. However, mean PAI-1 activity did not differ statistically significantly between variant carriers (n = 21; 18.00 ± 6.28 IU/mL) and non-carriers (n = 8; 15.77 ± 2.27 IU/mL) (*p* > 0.05), likely due to high interindividual variability within the carrier group. Nevertheless, the observed association suggests that this *VTN* variant cluster may influence fibrinolytic regulation, potentially contributing to LOAD pathogenesis through modulation of PAI-1 activity.

**Figure 2:**
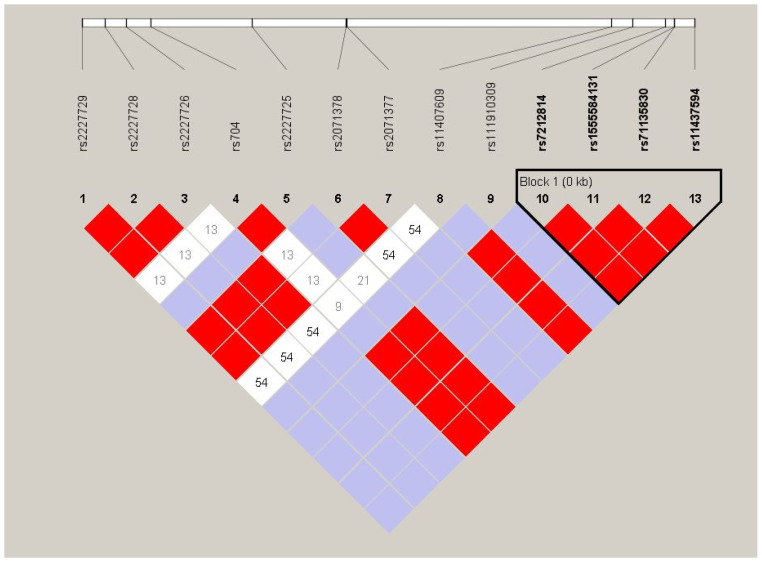
Haploview pairwise LD plot of 13 *VTN* variants in the combined cohort (19 LOAD cases, 10 controls). Cell color reflects D′/LOD: bright red, D′ = 1.0 with LOD ≥ 2; light blue, D′ = 1.0 with LOD < 2; white, D′ < 1.0. Numbers within cells indicate D′ × 100 (empty cells denote D′ = 1.0).

Linkage disequilibrium (LD) structure across the 13 genotyped SNPs was assessed using Haploview v4.2 in the combined cohort of 19 LOAD patients and 10 controls. Pairwise D′ values were computed under the confidence-interval algorithm of Gabriel et al. [17], and haplotype blocks were defined using the default criteria (with strong LD requiring an upper 95% confidence bound ≥ 0.98 and lower bound ≥ 0.70). The analysis identified a single haplotype block (Block 1) encompassing four SNPs at the 3′ end of the region (rs7212814, rs1555584131, rs71135830, and rs11437594), all of which were in complete pairwise LD (D′ = 1.0) (Figure 2). Notably, an excess co-occurrence of the minor alleles of rs704 and rs2227725 was observed, with the two variants displaying complete allelic association (D′ = 1.0); this non-random co-segregation suggests a shared ancestral haplotype background linking these two markers and may be of functional relevance given their location within the locus. Additional marker pairs—including rs2227729–rs2227728 and rs2071377–rs11407609—likewise showed high D′ values without reaching block-defining significance, likely reflecting the limited statistical power of the present sample. Intermediate D′ values (ranging from 0.09 to 0.54) were observed between the 5′ and central SNP clusters, indicating historical recombination events that partition the locus into distinct LD segments (Figure 2). These findings should be interpreted with caution, given the modest sample size (n = 29), which limits the precision of pairwise LD estimates and may underestimate block extent; replication in a larger independent cohort is warranted to confirm the observed haplotype architecture and to evaluate the potential biological significance of the rs704–rs2227725 association.

**Figure 3:**
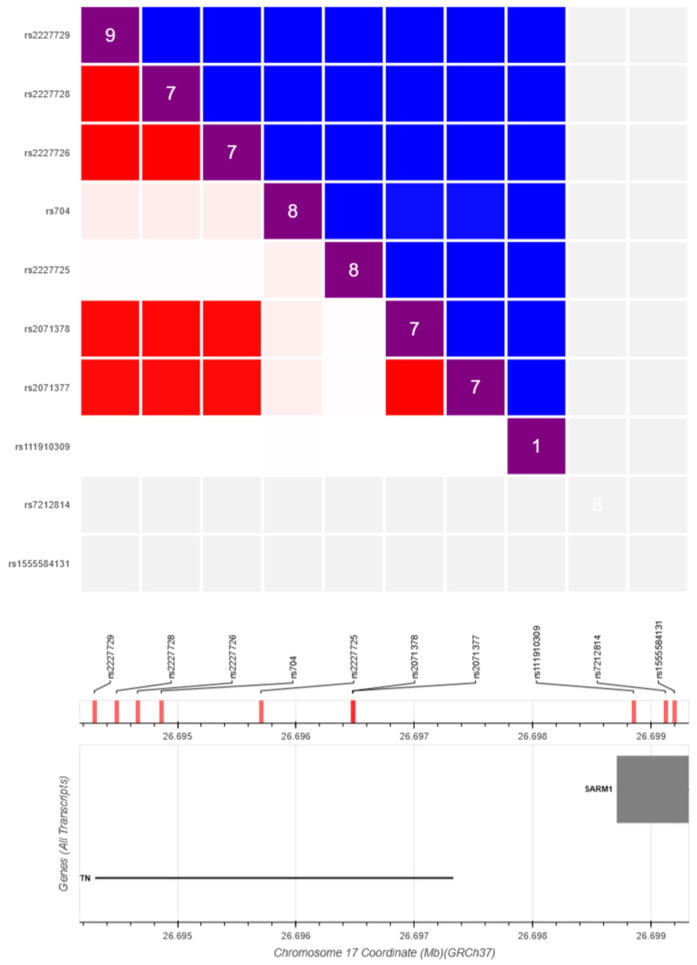
Pairwise LD of VTN variants in the 1000 Genomes EUR population (LDlink LDmatrix; GRCh37). Lower triangle: D′ (red); upper triangle: r^2^ (blue); diagonal: MAF × 100. The middle and bottom tracks show variant positions on chromosome 17. rs704 and rs2227725 show near-zero D′, indicating independent inheritance in healthy Europeans. (https://ldlink.nci.nih.gov/).

A striking divergence was observed when comparing the LD structure of our LOAD cohort with the 1000 Genomes European (EUR) reference panel. In the EUR population, rs704 (Thr400Met) and rs2227725 showed weak pairwise LD (D′ ≈ 0, light red/near-white in the LD matrix output), indicating that these two alleles are inherited largely independently in healthy European individuals (Figure 3). In sharp contrast, the two variants co-occurred in 16 of 19 AD patients (84.2%) in our cohort, while only 2 of 10 controls (20.0%) carried both alleles simultaneously (Fisher’s exact test, OR = 21.33, *p* = 0.001). This marked enrichment of the rs704–rs2227725 co-carrier state in LOAD cases—a combination that is not a recognized haplotype in the EUR reference—suggests a disease-associated allelic co-segregation pattern specific to this patient group. Although the small sample size warrants cautious interpretation, the magnitude of the effect and the absence of this co-occurrence signature in both population-level and local control datasets indicate that this variant pairing merits validation in larger, independently ascertained LOAD cohorts.

Targeted NGS results showed that 10 out of 19 patients (52.6%) carried the genetic risk factor for LOAD pathogenesis, APOE ε3/ε4 genotype. The remaining nine patients (47.4%) and ten cognitively normal age-matched control subjects carried the neutral APOE ε3/ε3 genotype. The PAI-1 activity of APOE ε3/ε3 carriers was 16.93 ± 4.05 IU/mL, and that of APOE ε3/ε4 carriers was 18.85 ± 8.16 IU/mL. The association between PAI-1 activity and APOE ε3/ε4 or APOE ε3/ε3 carriers was not statistically significant (*p* > 0.05). Thus, the increase in PAI-1 activity in LOAD patients is not related to APO ε4 status.

## 4. Discussion

In this study, a statistically significant increase in PAI-1 activity was observed in LOAD patients compared to the control group. We also identified an association between LOAD and both the T allele of the *VTN* rs704 (c.1199C>T) and *VTN * 5′-UTR variants. The *APOE ε4* allele frequency was 52.6% in the LOAD patients, which is consistent with its established role as a major genetic risk factor for LOAD. However, the lack of a statistically significant difference in mean PAI-1 activity between variant carriers and non-carriers (*p* > 0.05) suggests that this relationship may be due to high individual variability (SD = 6.28) within the carrier group rather than a linear dose–response effect. These findings support the hypothesis that *VTN * variants may contribute to LOAD pathogenesis by modulating PAI-1 activity, but they highlight the effect’s inherent complexity. Biasella et al. (2022) demonstrated that the *VTN* rs704 variant enhances PAI-1 binding affinity rather than its catalytic activity [14], suggesting that *VTN* variants may influence PAI-1 localization, stability, or cellular distribution rather than its activity.

In the present study, no significant difference in PAI-1 activity was observed across MMSE-based disease severity groups (*p* = 0.788). Although this finding may suggest that PAI-1 activity does not directly reflect current clinical disease stage, several important limitations must be considered before drawing firm conclusions. The sample size was very small, particularly in the severe group (n = 4), which substantially limited statistical power. The relatively high inter-individual variability (standard deviations ranging from ±4.4 to ±8.7) suggests that plasma PAI-1 activity may be influenced by factors other than disease severity, such as systemic inflammation, endothelial dysfunction, or comorbid conditions. Therefore, larger longitudinal studies with adequate statistical power are needed to definitively evaluate plasma PAI-1’s potential role as a biomarker. Future studies with larger cohorts and more comprehensive clinical data, including vascular comorbidities, inflammatory markers, and medication use, will be important to better delineate the relationship between plasma PAI-1 and disease-specific mechanisms.

The increased PAI-1 activity observed in our LOAD patients is consistent with the model of suppressed fibrinolysis in neurodegeneration. Elevated PAI-1 levels and suppressed fibrinolysis have been proposed as mechanisms that can start neurodegeneration in the central nervous system [18]. The role of fibrinolytic system components, including PAI-1 activity, in the pathophysiology of chronic complex diseases such as neurodegenerative diseases has been the focus of intense research [19,20]. Neurodegenerative diseases have complex pathophysiology involving inflammation [11]. The effects of PAI-1 and t-PA on the vascular and nervous systems present paradoxical and pleiotropic effects, such as permeability of the blood–brain barrier [21]. Clinical studies generally report PAI-1 antigen levels as a risk factor in vascular diseases [22]. However, in addition to the antigen levels, the stability and activity of PAI-1 have long been proven important [10]. Inflammation increases the activity and stability of PAI-1 [10,23]. We previously demonstrated the increased (nearly 43-fold) functional stability of PAI-1 in patients with thrombotic skin disorders [24]. Similarly, Eren et al. (2002) displayed coronary arterial thrombosis and alopecia areata in a transgenic expression animal model of conformationally stabilized active human PAI-1 [22]. Several environmental factors affect the stability of PAI-1. The interaction between proteases and other inflammatory proteins in neurodegenerative disease can affect the stability of the PAI-1.

Plasma PAI-1 levels predominantly reflect systemic vascular and inflammatory processes rather than central nervous system (CNS)-specific fibrinolytic activity. Nevertheless, emerging evidence suggests that systemic modulation of PAI-1 activity may still influence CNS pathology. For example, oral administration of TM5275, a small-molecule PAI-1 inhibitor, has been shown to reduce PAI-1 activity, increase tPA, uPA, and plasmin activity, and consequently decrease amyloid-β deposition in the hippocampus and cortex, along with improvements in cognitive function in APP/PS1 mice [25]. These findings support the notion that systemic PAI-1 activity may have indirect effects on brain fibrinolytic balance and neurodegenerative processes. Therefore, while plasma PAI-1 cannot be considered a direct surrogate marker of CNS PAI-1 activity, it may still provide complementary information regarding systemic pathways that are potentially linked to CNS pathology.

The genetic association with the *VTN* rs704 T allele offers a plausible mechanism for increased PAI-1 stability in LOAD. VTN is an inflammatory protein involved in cell adhesion and cancer progression [26]. Although active PAI-1 has a short half-life, binding to *VTN* not only stabilizes active PAI-1 but also enables it to regulate adhesion, migration, and extracellular matrix homeostasis through interaction with integrin αvβ3 [27,28,29]. The tertiary structure of VTN regulates fibrinolysis by increasing PAI-1 activity and protecting its active site from inactivation [30,31]. Additionally, the PAI-1- VTN interaction promotes microglial migration while inhibiting phagocytosis [30]. Recent studies on age-related macular degeneration (AMD) have provided important insights into the VTN rs704 variant. Although this variant does not affect the stabilization of active PAI-1, it does influence the binding capacity of VTN to PAI-1 [14]. Specifically, the T allele causes a stronger interaction between the AMD risk and PAI-1. The T allele enhances endogenous VTN expression and is associated with a modestly increased binding affinity for PAI-1, leading to a stronger interaction between PAI-1 and AMD risk [13,14]. In the present study, the T allele frequency was higher in LOAD patients compared to controls, suggesting a strong association with disease susceptibility.

There are conflicting reports on PAI-1 levels in AD. PAI-1 is associated with Aβ accumulation, and knocking out the *SERPINE1* gene or adding PAI-1 inhibitors reduces Aβ accumulation in the mouse model of AD [25,32]. Serum PAI-1 levels positively correlate with cognitive impairment in AD patients [33], and PAI-1 levels increase in AD patients while serum t-PA levels remain unchanged [34]. In contrast, other studies have demonstrated that PAI-1 levels decrease in both preclinical and clinical AD [35,36]. These conflicting results may be partly explained by the fact that, in addition to PAI-1 concentration, the stability of its active form is also functionally important. In complex diseases, there can be a mismatch between serpin concentration and activity. The present study indicates that PAI-1 activity is prolonged in LOAD patients. We propose that VTN and neuroinflammation may potentially increase PAI-1 activity in neurodegenerative diseases.

Age is a primary risk factor for neurodegenerative diseases, yet their pathophysiology remains incompletely understood. Several lines of evidence suggest a causal role for PAI-1 in aging as components of the fibrinolytic system, including PAI-1, which can modulate the aging process. Studies indicate that cellular senescence and premature aging are associated with increased PAI-1 expression [37]. In line with this, the present study demonstrates that PAI-1 activity—a potential mediator of central nervous system senescence and brain aging—is increased in LOAD. The significance of this finding is underscored by the well-established role of PAI-1 in cellular senescence and aging, the primary risk factor for LOAD [37,38]. Furthermore, carriers of the null *SERPINE1* mutation exhibit significantly longer leukocyte telomere length, lower fasting insulin levels, lower prevalence of diabetes mellitus, and a longer lifespan [38].

The absence of a direct correlation between PAI-1 activity and specific *VTN * or *APOE* genotypes points to the multifaceted regulation of this pathway, likely involving additional genetic and environmental factors. Although we observed an association between specific VTN variants and altered PAI-1 activity, we cannot infer causation or a direct molecular mechanism. PAI-1 is likely at the crossroads of disturbed metabolism and neurodegeneration. At present, intravenous thrombolysis is the only way to activate fibrinolysis in humans, but oral PAI-1 inhibitors have been in phase 1-3 trials for other indications [39] and may represent a future therapeutic option for neurodegenerative diseases [40]. Future functional studies—including VTN protein expression and purification, PAI-1 activity assays in VTN-overexpressing or knockdown cellular models, and investigation of the VTN-PAI-1 interaction interface—are essential to establish whether VTN variants directly modulate PAI-1 regulation and thereby influence LOAD pathogenesis.

## 5. Conclusions

In conclusion, our data position PAI-1 activity, potentially modulated by *VTN * genetics, at the crossroads of fibrinolysis, neuroinflammation, and aging in LOAD. The development of oral PAI-1 inhibitors currently in clinical trials for other indications [41] opens a novel therapeutic avenue for neurodegenerative diseases. Future studies with larger cohorts are required to assess the putative epistasis between *VTN*, *SERPINE1*, and *APOE* genes as well as the additive effects of each risk allele on LOAD susceptibility and specific phenotypic subsets. Collectively, these findings highlight the need to consider PAI-1 activity not merely as a peripheral vascular marker but as a potential central mediator of LOAD pathogenesis.

## Figures and Tables

**Table 1:** Characteristics of the study population. MMSE, mini-mental state examination; CDR, clinical dementia rating scale; SD, standard deviation.

Characteristics	LOAD, n = 19	Controls, n = 10
Age, mean ± SD	77.84 ± 6.13	77.60 ± 1.58
Female/male	9/10	5/5
MMSE score		
>20, mild (n)	22.38 ± 1.51 (8)	
10–19, moderate (n)	14.43 ± 2.51 (7)	Normal
<10, severe (n)	6.00 ± 3.83 (4)	
CDR score	n	n
0, normal	0	10
1, mild	6	0
2, moderate	6	0
3, severe	7	0

**Table 2:** Frequencies of SERPINE1 and VTN gene variants in LOAD patients and age-matched cognitively normal control subjects.

*SERPINE1* (*PAI-1*), chr 7	Rs Number	Ref/SNP	Location	Amino Acid Change	LOAD/Control	Fisher’s *p*-Value	Bonferroni Adjusted *p*-Value	GnomAD
100771717	rs6092	G/A	Exon 2	p.Ala15Thr	4/0	0.27	1.00000	0.09
100771723	rs6090	G/A	Exon 2	p.Val17Ile	1/0	1	1.00000	0.29
100776931	rs2227684	G/A	Intron		10/2	0.13	1.00000	0.41
100777197	rs139464072	C/T	Intron		1/0	1	1.00000	0.0026
100780903	rs41334349	C/T	3′-UTR		1/0	1	1.00000	0.0066
100781084	rs11178	T/C	3′-UTR		8/0	0.027	0.59400	0.44
100781445	rs7242	T/G	3′-UTR		1/1	1	1.00000	0.45
100781711^ 100781712	rs41423845	-/CGCGCCCCC	3′-UTR		19/2	0.0001	0.00220	0.87
100781615	rs1050813	G/A	3′-UTR		7/1	0.20	1.00000	0.14
***VTN*, chr17**								
26698673^ 26698674	rs11407609	-/C	5′-UTR		7/1	0.20	1.0000	0.67
26698851	rs111910309	C/T	5′-UTR		0/1	0.35	1.0000	0.0014
26699121	rs7212814	C/G	5′-UTR		19/2	0.0001	0.0022	1
26699195^ 26699196	rs1555584131	-/C	5′-UTR		19/2	0.0001	0.0022	1
26699199^ 26699200	rs71135830	-/C	5′-UTR		19/2	0.0001	0.0022	0.99
26699367^ 26699368	rs11437594	-/C	5′-UTR		19/2	0.0001	0.0022	1
26694296	rs2227729	A/G	5′-UTR		3/0	0.53	1.0000	0.17
26694483	rs2227728	A/G	Exon 8	p.Asn448=	3/0	0.53	1.0000	0.11
26694661	rs2227726	G/A	5′-UTR		3/0	0.53	1.0000	0.15
26694861	rs704	G/A	Exon 7	p.Thr400Met	16/2	0.001	0.0220	0.51
26695704	rs2227725	A/G	intron		19/2	0.0001	0.0022	0.96
26696477	rs2071378	A/G	5′-UTR		3/0	0.53	1.0000	0.23
26696482	rs2071377	C/T	5′-UTR		3/0	0.53	1.0000	0.23

## Data Availability

The original contributions presented in the study are included in the article, further inquiries can be directed to the corresponding author.

## Abbreviations

The following abbreviations are used in this manuscript:

α-SYN	Alpha-synuclein
AMD	Age-related maculodegeneration
APOE	Apolipoprotein E
LOAD	Late-onset Alzheimer’s disease
DSM-IV	Diagnostic and Statistical Manual of Mental Disorders-IV
EDTA	Ethylenediaminetetraacetic acid
IU	International unit
IQR	Interquartile range
MMSE	Mini Mental Status Exam
NGS	Next generation sequencing
PAI-1	Plasminogen activator inhibitor-1
pNA	Para-nitroaniline
t-PA	Tissue plasminogen activator
u-PA	Urokinase-type plasminogen activator
VTN	Vitronectin
